# Novel Oncologic Strategies in Osteosarcoma Management: Maximizing Impact, Minimizing Harm

**DOI:** 10.15190/d.2025.15

**Published:** 2025-09-30

**Authors:** Taha Kassim Dohadwala, Rabab Hunaid Abbas, Sharin Mary Siji, Soumiya Nadar, Sakina Sakina, Naman Jairath, Hetvi Ashwinkumar Patel, Tinatin Zurashvili

**Affiliations:** ^1^Department of Medicine, Faculty of Medicine, David Tvildiani Medical University, Tbilisi, Georgia; ^2^Department of Medicine, Faculty of Medicine, Tbilisi State Medical University, Tbilisi, Georgia; ^3^Department of Medical Biochemistry, David Tvildiani Medical University, Tbilisi, Georgia

**Keywords:** Doxorubicin, Osteosarcoma, Chemotherapy, Cardiotoxicity, Drug Delivery Systems, Targeted Therapy, Efficacy Enhancement, Toxicity Reduction.

## Abstract

Doxorubicin (DOX), an anthracycline antibiotic, is pivotal in managing osteosarcoma (OS). Although a cornerstone in the multimodal treatment regimen, its usage is limited by its associated toxicities, such as cardiotoxicity, myelosuppression, and neurotoxicity. This review provides an in-depth discussion of the emerging research aimed at enhancing the overall efficacy and mitigating the undesired side effects of DOX in treating OS. We explore the various drug delivery systems, including polymer-based injectable hydrogels, hydroxyapatite-based systems, and various nanoparticle-based systems such as calcium carbonate nanocrystals and cerium-substituted hydroxyapatite. We also discuss various innovative combination therapies, such as pegylated liposomal DOX with cisplatin and DOX with platinum nanoparticles. Moreover, emerging research into light-sensitive nano-micelles have been highlighted. These methods improve DOX's cytotoxicity and potentially reduce the need for high systemic doses and their associated side effects. The review aims to highlight the promising future in OS treatment by integrating these methodologies to maximize the therapeutic action of DOX and reduce its systemic side effects.

## SUMMARY

*1. Introduction*

*2. Mechanisms of Action of Doxorubicin*

*2.1 DNA adduct formation and intercalation*

*2.2 Topoisomerase II inhibition*

*2.3 Oxidative Stress*

*3. Side Effects and Challenges of Doxorubicin Therapy*

*4. Mechanisms of Doxorubicin Resistance in Osteosarcoma*

*5. Advancements in Overcoming Doxorubicin Toxicities and Enhancing Treatment Efficacy*

*5.1 Polymer-Based Drug Delivery Systems*

*5.2 Hydroxyapatite-Based Delivery Systems*

*5.3 Pegylated Liposomal DOX (PEG-LD)*

*5.4 Platinum Nanoparticles (PtNPs) Combined with DOX*

*5.5 Calcium Phosphate-Phosphorylated Adenosine Microspheres*

*5.6 Nanoparticle-Based Drug Delivery Systems*

*6. Challenges and Future Directions*

*7. Conclusion*

## 1. Introduction

Primary bone cancers are rare malignant neoplasms, accounting for less than 0.2% of all malignancies worldwide^^1^^. Osteosarcoma (OS), the most commonly diagnosed primary bone cancer in adolescents, exhibits two age-specific peaks: the first occurs during the second decade of life (ages 15-19), and the second appears after the sixth decade^^2,3^^. Although relatively rare, osteosarcoma presents a unique challenge due to its aggressive nature and a predilection for the younger population, with an average incidence rate of 1.9 per million in males and 1.36 per million in females^^4^^. The majority of osteosarcomas are restricted to the metaphysis of the distal femur, proximal tibia, and proximal humerus, with affected individuals genetically predisposed to hereditary conditions, including hereditary retinoblastoma and Li-Fraumeni syndrome^^5,6^^. Owing to its aggressive nature and propensity to metastasize to the lungs, management of OS requires a multimodal approach involving a combination of systemic chemotherapy and surgical resection^^7,8^^.

**Figure 1:**
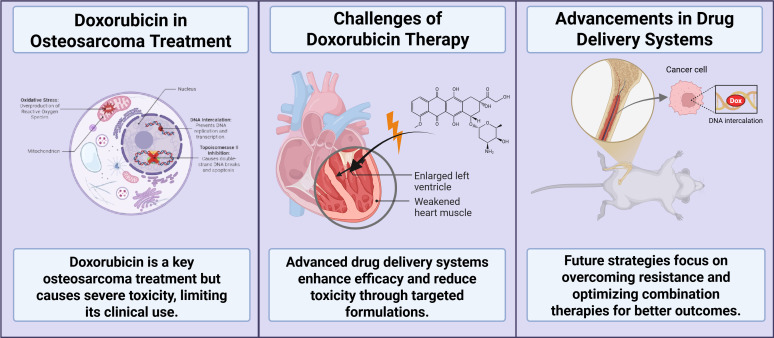
Figure 1. Overview of Doxorubicin Use, Challenges, and Delivery Advances in Osteosarcoma Treatment. Created with BioRender.com

Despite the current advancements made in surgical management and chemotherapy protocols, the overall prognosis of osteosarcoma remains unfavorable, particularly in cases of metastasis and recurrence^^9,10^^.

Central to the chemotherapy regimen is doxorubicin (DOX), an anthracycline, which plays a crucial role in treating various solid and hematologic malignancies, including osteosarcoma^^11^^. The primary mechanism of action of this drug involves the intercalation of DNA strands, causing breaks, interference with topoisomerase II enzyme (TOP2) activity, and generation of free radicals, all of which allow DOX to penetrate the malignant cell barrier, leading to cell death^^12^^. However, one of the potential drawbacks of DOX therapy is drug-induced cardiotoxicity, a serious and potentially fatal adverse effect with an incidence of late cardiotoxicity of approximately 1.7%^^11,13^^.

Several reviews have explored the role of DOX in OS, often focusing on either its mechanism of action or broad nanotechnology-based approaches. In contrast, this review integrates molecular mechanisms of resistance with an updated synthesis of targeted delivery strategies reported over the past decade, emphasizing their translational relevance. It also highlights emerging approaches aimed at enhancing therapeutic efficacy, with the ultimate goal of improving outcomes for patients with OS. (Figure 1).

## 2. Mechanisms of Action of Doxorubicin

**Figure 2:**
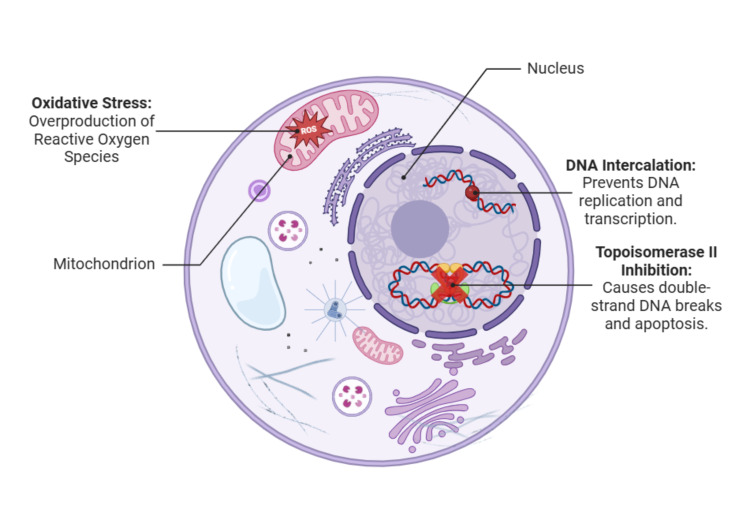
Figure 2. Mechanisms of Action of Doxorubicin (DOX) cytotoxicity - DNA intercalation, TOP2 inhibition, and ROS-induced mitochondrial dysfunction leading to apoptosis in osteosarcoma cells. Created with BioRender.com

DOX, an anthracycline antibiotic, is characterized by its aliphatic side chains, which play an essential role in its binding to the DNA helix. It is known for its cytotoxic effects, achieved through several mechanisms, three of which are particularly well-documented (Figure 2)^^14^^.

### 2.1 DNA adduct formation and intercalation

This mechanism involves the formation of DNA adducts and intercalation into DNA strands. DOX binds to guanine in G-C base pairs through hydrogen bonds, causing DNA untwisting and positive supercoiling^^15-18^^. This intercalation process is associated with an increased turnover of nucleosomes around the promoter regions of active genes^^18^^. To validate this mechanism, the CATCH-IT (Covalent Attachment to Tags to Capture Histones and Identify Turnover) method was developed to measure nucleosome turnover and link it to DOX's cytotoxic effects. In a mouse squamous cell carcinoma model, two cell lines were compared before and after DOX treatment. The results showed increased nucleosome turnover around promoter regions of active genes, which correlated with the observed cytotoxic effects^^19^^. Additionally, DOX forms DOX-DNA adducts, activating the DNA damage response pathway. These adducts release formaldehyde as a byproduct. This facilitates the formation of covalent bonds between DOX and guanine base pairs on the same strand, as well as hydrogen bonds with guanine on the opposite strand. The resulting interstrand cross-links hinder DNA repair processes and ultimately induce cell death^^20,21^^. The eukaryotic cell's dependence on TOP is fundamental to several nuclear processes, including DNA replication, transcription, and the preservation of DNA sequence integrity.

### 2.2 Topoisomerase II inhibition

DOX's mechanism of action involves inducing breaks in the DNA structure, which leads to upregulation of TOP2 activity. This up-regulation increases the formation of TOP2-DNA covalent complexes, effectively "poisoning" the enzyme and blocking transcription and replication, ultimately leading to apoptosis^^22,23^^. Notably, DOX at doses below 1 µM can covalently capture TOP2 at double-strand break sites, inhibiting DNA religation^^24,25^^. The discovery of Ataxia Telangiectasia and Rad3-related (ATR), a protein kinase structurally related to phosphoinositide 3-kinase, has shed light on its role in the replication stress response. ATR may respond to TOP2 inhibition by halting or delaying DNA polymerase activity, thereby preventing replication in cancer cells^^26,27^^.

### 2.3 Oxidative Stress

Reactive oxygen species (ROS) play a dual role in the body, acting as messengers at low concentrations but causing DNA damage at high concentrations^^28^^. DOX is implicated in the overproduction of ceramide, which contributes to ROS generation, thereby increasing ROS levels^^29^^. Moreover, ceramide triggers the release of pro-apoptotic proteins from the mitochondria, a precursor to apoptosis^^30-32^^. DOX is capable of directly binding to cardiolipin located on the inner mitochondrial membrane, leading to an excess of ROS production^^33-35^^. Excessive accumulation of DOX disrupts the electron transport chain (ETC), particularly by interfering with complex I. This disruption increases ROS production, causes mitochondrial damage, and ultimately induces cell death. DOX can bind to complex I, disrupting electron transfer from NAD(P)H to NAD(P)+ and diverting electrons to DOX instead^^36,37^^.

By providing an overview of the various mechanisms of action of DOX, including DNA intercalation, TOP2 inhibition, and oxidative stress, we highlighted the key cytotoxic actions of DOX against OS cells.

## 3. Side Effects and Challenges of Doxorubicin Therapy

**Figure 3:**
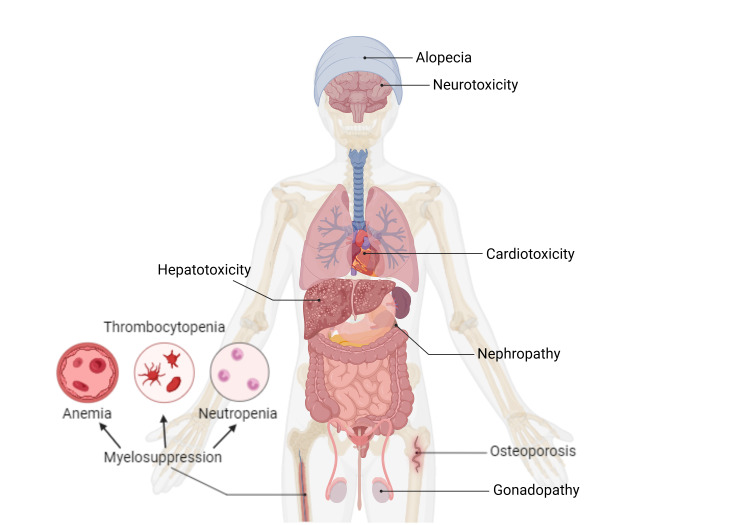
Comprehensive Overview of Doxorubicin Side Effects: Understanding the Range of Adverse Reactions. This figure emphasizes the various side effects associated with doxorubicin. Created with BioRender.com.

Although DOX serves as the key element in the primary treatment for various malignancies, including OS, its use is associated with several significant side effects (Figure 3). While numerous adverse effects of doxorubicin have been reported, cardiotoxicity remains the most clinically significant and dose-limiting complication. Acute cardiotoxicity includes reversible myopericarditis, left ventricular dysfunction, and DOX-induced arrhythmias, which affect up to 26% of patients. DOX-induced cardiomyopathy is a chronic complication that can occur from a few months to up to twenty years after the end of treatment^^11^^. Cardiotoxicity is strongly linked to mitochondrial ROS overproduction. Cardiomyocytes, with their high ATP demand, are particularly vulnerable to cardiovascular diseases due to the inability of cardiac mitochondria to meet energy requirements^^35,38^^. Additionally, DOX’s toxic effects on mitochondria include the production of superoxides, reactive nitrogen species, and intracellular Ca^^2+^^ dysregulation, which further contribute to ROS production. DOX also activates both intrinsic and extrinsic apoptosis pathways in cardiomyocytes^^39^^.

Mitigation strategies include early risk stratification and monitoring using echocardiography and biomarkers, limiting cumulative doses (<450-550 mg/m²), and prolonging infusion times (≥6 hours) to reduce peak drug levels and lower odds of LV dysfunction^^40,41^^. Pharmacologic protection with agents like dexrazoxane^^42^^, beta-blockers^^43^^, RAAS inhibitors^^44^^, and statins^^45,46^^, and employing less cardiotoxic liposomal and exosomal formulations^47^ further preserves cardiac function^^40^^. Lifestyle modifications such as smoking cessation and tailored exercise contribute to lowering risk^^40^^. Novel therapies targeting mitochondrial function, apoptosis pathways, and genetic susceptibility are under investigation to further enhance cardioprotection^^40,48^^.

DOX induces myelosuppression by selectively targeting bone marrow stem cells (BMSCs), particularly those expressing the ABCG2 transporter, leading to significant reductions in their numbers and function, which manifests as decreased white blood cell (WBC) count and overall bone marrow suppression^^49^^. Neutropenia increases infection risk and hospitalization rates, while anemia often presents with debilitating fatigue, which impairs physical and emotional functioning. Thrombocytopenia heightens bleeding risk and anxiety^^50^^. Preventive strategies include supportive therapies like granulocyte colony-stimulating factor (G-CSF) to promote neutrophil recovery, erythropoietin, and red blood cell (RBC) transfusions for anemia. However, these traditional supportive care carries side effects such as bone pain, thromboembolism, and transfusion reactions^^51,52^^. Newer therapeutic agents show promise, including trilaciclib, which is a CDK4/6 inhibitor, offering proactive myeloprotection by temporarily arresting hematopoietic stem cell division before chemotherapy, significantly reducing grade 3-4 cytopenias and improving patient-reported outcomes^^53^^.

Gonadotoxicity is another concern, as DOX can cause DNA double-strand breaks, leading to follicle depletion in females and impaired spermatogenesis in males^^54-56^^. Patients undergoing DOX chemotherapy may experience cognitive impairments such as memory loss, reduced concentration, and difficulty multitasking. Sperm and oocyte preservation are feasible and increasingly accessible, while gonadal tissue cryopreservation remains experimental^^57^^.

Hepatocytes are also impacted by DOX accumulation and ROS production, which overcomes the liver’s regenerative capacity and impairs ABC transporters, disrupting metabolic pathways related to cell proliferation and death^^58^^. DOX is also associated with nephropathy, with animal studies showing structural changes in the kidneys, including effects on glomerular capillaries, compromised podocyte integrity, and degenerative changes in the proximal convoluted tubule^^59^^.

Studies on DOX-treated mice have demonstrated several markers of central nervous system toxicity. These include increased protein and lipid oxidation in the brain, elevated TNF-α levels, glial cell activation in the cortex and hippocampus, mitochondrial dysfunction, oxidative stress, cytochrome C release, and enhanced caspase-3 activity^^60^^. Additionally, DOX has been shown to affect bone structure in mouse models, with treated mice displaying reduced trabecular bone mass. Co-culture studies with mouse BMSCs have demonstrated a dose-dependent reduction in osteogenic differentiation, suggesting that DOX may also contribute to osteoporosis^^61^^.

## 4. Mechanisms of Doxorubicin Resistance in Osteosarcoma

**Figure 4:**
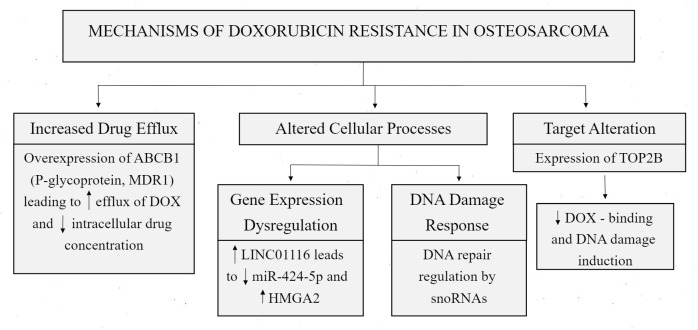
Mechanisms of Doxorubicin Resistance: Exploring Genetic and Molecular Factors. This figure highlights the focus on the various mechanisms through which resistance to doxorubicin can develop.

Although survival rates have improved with the introduction of multimodal regimens that combine chemotherapy and surgery, prognosis remains poor for many patients. Approximately 40-45% develop resistance to DOX, largely due to acquired drug resistance^^62^^. Several mechanisms contribute to DOX resistance in OS (Figure 4), including enhanced drug efflux, altered signal transduction, target gene mutations, changes in drug metabolism, inhibition of cell death, tumor immune responses, and increased DNA repair capacity^^63,64^^.

Several mechanisms have been implicated in the resistance of DOX in OS. One of the most pertinent is the ATP-binding cassette (ABC) transporter-mediated drug efflux. Here, the ABCB1 (P-glycoprotein, MDRI) overexpression has been reported to limit the drug's intracellular accumulation and cytotoxicity^^65,66^^. Further, ABCA1, a distinct ABC transporter, aids in the treatment of osteosarcoma by removing isopentenyl pyrophosphate, a substance that attracts immune T-cells capable of fighting tumors. However, OS cells with elevated ABCB1 levels show reduced ABCA1 expression, rendering them resistant to both medication and immune response^^66,67^^.

In addition to changes in enzyme function, the development of drug resistance can also result from pathogenic elevations in target enzymes or a reduced binding affinity of the drug to the target enzyme. The TOP2 enzyme is a nuclear protein involved in the DNA replication process. TOP2 forms a homodimer and functions by cleaving double-stranded DNA (dsDNA), passing a second DNA duplex through the break, and then re-ligating the strands. This activity is essential for resolving DNA supercoiling and entanglements, making TOP2 necessary for DNA replication. TOP2 has been recognized as a target of DOX. Two isoforms of TOP2 are found in humans: TOP2α and TOP2β. Reduced expression of TOP2β has been associated with DOX resistance in several tumors, including osteosarcoma. Specifically, DOX-resistant OS cells exhibit lower levels of TOP2β compared to DOX-sensitive cells^^63^^.

Studies show that LINC01116, an oncogene for multiple cancers, including OS, was upregulated in the DOX-resistant OS cell line MG-63/Dox. Studies have shown epithelial-mesenchymal transition (EMT)'s relationship with tumor metastasis and drug resistance in malignant cells; therefore, regulation of EMT has become a novel approach for anti-tumor therapies. Investigation into EMT in OS cells revealed that elevated LINC01116 levels led to the suppression of miR-424-5p and the downstream overexpression of HMGA2, promoting DOX-resistance in OS cells^^68^^.

Long non-coding RNAs (lncRNAs) have also been a focus in cancer pathogenesis, including OS. Many antisense lncRNAs are implicated in metabolic processes by regulating endogenous gene expression.

lncRNA FOXC2-AS1 and its antisense transcript FOXC2 are up-regulated in DOX-resistant OS cell lines and tissues, promoting poor prognosis in OS cells both in vitro and in vivo. It was concluded that lncRNA FOXC2-AS1 increases the expression of transcription factor FOXC2, which in turn enhances the expression of ABCB1^^69^^.

Additionally, small nucleolar RNAs (snoRNAs) have been identified as markers of DOX resistance in OS cells. These snoRNAs contribute to resistance by regulating the expression of genes involved in DNA damage sensing, DNA repair, ribosome biogenesis, and cell proliferation. Targeting snoRNAs or the genes they influence may offer new therapeutic strategies for overcoming chemoresistance in OS^^70^^.

A major challenge in cancer treatment is the development of drug resistance, which often leads to cancer relapses. While there has been progress, only a few studies have demonstrated success in overcoming DOX resistance. By targeting the ABCB1 gene in MDR-OS cells, the CRISPR-Cas9 system blocks the expression of the transporter protein P-glycoprotein. This modification has shown promise in reversing DOX resistance^^71^^. Additionally, the bisindolic alkaloid voacamine has been studied for its efficiency in enhancing the cytotoxicity of DOX on MDR cells by increasing drug retention and intranuclear location^^72^^. Moreover, clinical modulation of the underlying mechanism of LINC01116’s upregulation in the DOX-resistant OS cell line MG-63/Dox and its influence on the EMT process could be used as a promising chemosensitizing strategy for the treatment of OS^^68^^. However, the complexity of DOX resistance in OS requires further research and interventions.

## 5. Advancements in Overcoming Doxorubicin Toxicities and Enhancing Treatment Efficacy

DOX is a potent anthracycline chemotherapy agent and has been a cornerstone in the treatment regimen against various cancers, including bone cancer. However, the success of DOX hinges on achieving precise concentrations in tumor cells post-systemic administration. Failure in these concentrations leads to life-threatening complications such as cardiomyopathy, congestive heart failure, mucosal tissue damage, bone marrow toxicity, and alopecia. This comprehensive understanding has paved the way for research on improving DOX’s effectiveness in OS. Herein, we review the latest advancements in DOX delivery systems, including pre-clinical and clinical studies, to better understand its potential for OS treatment (Table 1).

### 5.1 Polymer-Based Drug Delivery Systems

Polymer-based drug delivery systems, especially injectable hydrogels, are increasingly popular for bone cancer treatment due to their minimally invasive application, biodegradability, and sustained drug release. Thermosensitive hydrogels enable the co-delivery of DOX and anticancer genes, enhancing efficacy by solidifying at body temperature after injection^^73^^. In a preclinical study, Ma et al. developed a poly(L-lactide-co-glycolide)-poly(ethylene glycol)-poly(L-lactide-co-glycolide) triblock copolymer (PLGA-PEG-PLGA) hydrogel for the co-delivery of DOX, CDDP, and MTX, achieving synergistic effects and improved tumor inhibition in OS cell models^^74^^. Another approach involves zeolite-based systems to modulate drug release. In a preclinical study, ZSM-5 zeolite nanodisks effectively delivered DOX to induce apoptosis in cancer cells^^73^^.

### 5.2 Hydroxyapatite-Based Delivery Systems

**Table 1:** Summary of DOX Delivery Systems for Osteosarcoma Treatment

Delivery System	Research Phase	Study/Research	Key Findings
Polymer-Based Drug Delivery Systems	Preclinical Study	Injectable hydrogels for co-delivery of DOX and anticancer genes	Enhanced efficacy through sustained drug release and minimally invasive application(73)
PLGA-PEG-PLGA hydrogel for DOX, CDDP, and MTX	Synergistic effects and improved tumor inhibition in OS models(74)		
Hydroxyapatite-Based Delivery Systems	Preclinical Study	DOX delivery via nano-HA (nHA) and micro-HA (mHA) particles	Significant tumor reduction and high DOX binding rates in OS models(75)
Pegylated Liposomal DOX (PEG-LD)	Clinical Study	Phase I study combining PEG-LD and cisplatin in metastatic and recurrent OS	MTD at 50 mg/m2 with acceptable safety profile and promising clinical activity(76)
Platinum Nanoparticles (PtNPs) Combined with DOX	Preclinical Study	PtNPs combined with low-dose DOX for U2OS cells	Significant anticancer activity through increased oxidative stress and apoptosis(77)
Calcium Phosphate-Phosphorylated Adenosine (CPPA) Microspheres	Preclinical Study	Fast microwave-assisted solvothermal method for creating CPPA structures	High DOX encapsulation efficiency, pH-responsive release, and enhanced osteogenesis(78)
Nanoparticle-Based Drug Delivery Systems	Preclinical Study	Ag2O-MBG NPs for DOX encapsulation	High encapsulation efficiency, pH-dependent release, and significant inhibition of OS cells(79)
Light-responsive nano-micelle systems (Poly-Dox-M)	Targeted DOX release at the tumor site with minimal systemic toxicity(80)		
Calcium-carbonate nanocrystals for DOX delivery	pH-dependent release and significant inhibition of MG-63 OS cells(81)		
Cerium-substituted hydroxyapatite (Ce-HA) nanoparticles	Sustained DOX release and promising anticancer activity with reduced systemic toxicity		
Hybrid Nanogels for Co-Delivery of CDDP and DOX	Synergistic antitumor effects, optimized biodistribution, and reduced long-term toxicity(82)		

This preclinical study investigated the effectiveness of DOX delivered via hydroxyapatite (HA) particles for OS treatment. Scanning electron microscopy (SEM) revealed rod-shaped nano-HA (nHA) and spherical micro-HA (mHA), both with high purity confirmed by X-ray diffraction (XRD). In vitro experiments showed strong DOX binding to HA, increasing with nHA amounts up to 40 mg and DOX doses up to 800 μg, achieving a 91.8% binding rate. The binding was driven by electrostatic interactions with calcium ions and was minimally affected by serum proteins. Intracellular studies confirmed nHA+DOX uptake and pH-dependent release, showing significant biological effects on ATP synthesis, cell migration, and viability. In vivo binding affinity was further confirmed by implanting nHA and mHA in the abdominal muscle pouch of rats. It showed higher fluorescence signals compared to collagen sponge controls and surrounding muscle, indicating 2-3 times more DOX recruitment. In an osteosarcoma xenograft model, HA-delivered DOX significantly reduced tumor progression. The highest binding was observed at 50.5% for 48-hour exposure and 91.8% for 800 μg DOX, highlighting the potential of this targeted delivery system to enhance DOX therapeutic efficacy^^75^^.

### 5.3. Pegylated Liposomal DOX (PEG-LD)

A phase I clinical study aimed to determine the maximum tolerated doses (MTDs) and dose-limiting toxicities (DLTs) of pegylated liposomal DOX (PEG-LD) combined with CDDP in patients with metastatic and recurrent OS. This study was designed considering the reduced cardiac toxicity of PEG-LD compared to traditional DOX. Patients received PEG-LD at doses of 40, 50, or 60 mg/m2 on day 1 of each 21-day cycle, with CDDP at a fixed dose of 100 mg/m2, following a 3+3 dose escalation approach. Among the 15 patients enrolled, including nine who had previously received DOX. The MTD of PEG-LD was established at 50 mg/m2, with neutropenic fever and stomatitis as DLTs. The primary adverse event(AE) was myelosuppression, while common non-hematological AEs included vomiting, hypoproteinemia, stomatitis, and transient sinus arrhythmia. Grade 3-4 toxicities such as neutropenia, leukopenia, thrombocytopenia, anemia, and stomatitis were observed, but all AEs were managed with supportive treatment. The overall response rate was 13.3%, with a disease control rate of 66.7%. Among patients without prior DOX exposure, one achieved a partial response, and five had stable disease. The study concluded that PEG-LD at 50 mg/m2 combined with CDDP at 100 mg/m2 has an acceptable safety profile and promising clinical activity in advanced OS, warranting further evaluation in phase II trials^^76^^.

### 5.4. Platinum Nanoparticles (PtNPs) Combined with DOX

A preclinical study found that platinum nanoparticles (PtNPs) at 10 μg/mL, combined with low doses of DOX at 1.0 μg/mL, exhibit significant anticancer activity in U2OS cells. The combination reduced cell viability and proliferation, increased oxidative stress, and induced mitochondrial dysfunction and DNA damage. PtNPs enhanced the effects of DOX by promoting oxidative stress and apoptosis, suggesting a synergistic interaction. These findings indicate that PtNPs combined with DOX could be an effective therapeutic strategy for OS, though further research is needed to understand the underlying molecular mechanisms^^77^^.

### 5.5. Calcium Phosphate-Phosphorylated Adenosine Microspheres

In this preclinical investigation, researchers developed microspherical structures using a fast microwave-assisted solvothermal method (110 °C, 10 min) based on calcium phosphate-phosphorylated adenosine (CPPA). The CPPA system exhibited a porous and hollow structure, enabling a high encapsulation efficiency of DOX (approximately 42.3%) and demonstrating pH-responsive drug release characteristics. In vitro and in vivo studies revealed that the loaded CPPA system exhibited a therapeutic effect on osteosarcoma cells. Additionally, it stimulated the osteogenesis of human bone marrow-derived mesenchymal stem cells (BM-MSCs). This effect is mediated by enhancing the expression of specific markers such as ALP activity, osteocalcin (OCN), osteopontin (OPN), and collagen type I^^78^^.

### 5.6. Nanoparticle-Based Drug Delivery Systems

All the studies discussed in this section are preclinical studies focusing on various nanoparticle-based drug delivery systems for DOX in OS treatment.Silver oxide doped mesoporous bioactive glass nanoparticles (Ag20-MBG NPs) for Controlled Release - This study analyzed the encapsulation efficiency and release kinetics of DOX encapsulated within Ag2O-MBG NPs, highlighting its potential therapeutic efficacy against OS. With an encapsulation efficiency of 84%, DOX within the Ag2O-MBG NPs demonstrated a pH-dependent release pattern. Specifically, a release of 93% was observed over two weeks at a slightly acidic pH of 6.4. This controlled release profile suggests the potential for sustained therapeutic efficacy while minimizing systemic toxicity. Moreover, the study demonstrated notable inhibitory effects on the viability of MG-63 OS cancer cells, indicating the promise of Ag2O-MBG NPs as an effective strategy for bone tissue regeneration and bone cancer treatment. However, further investigations are warranted to elucidate the in vivo efficacy and safety profile of this nanoparticle-based delivery system^^79^^.Light-responsive Nano-Micelle Systems - This study addresses the challenges associated with systemic toxicity and limited tumor targeting. It presents a novel approach to DOX delivery using a light-responsive nano-micelle drug delivery system, termed Poly-Dox-M. It incorporates polyethylene glycol (PEG) to ensure stable nanostructure in the bloodstream and enhance tumor targeting via the enhanced permeability and retention (EPR) effect. Importantly, upon exposure to light irradiation, the micelles undergo a rapid structural change at the tumor site, shedding the PEG shell and releasing the encapsulated DOX. This targeted drug release mechanism enhances DOX uptake by tumor cells, thereby increasing chemotherapy efficacy while minimizing systemic toxicity. It offers sensitivelight-responsive characteristics, enhanced anti-cancer effects, a favorable biosafety profile, and efficient cellular uptake. An experimental study assessed the in vitro release kinetics of DOX from drug-loaded calcium carbonate nanocrystal suspensions. These suspensions, containing approximately 10 mg of nanoparticles, were prepared in 50 mL of PBS buffer at varying pH levels. Measurement of DOX concentration at specific intervals revealed a controlled and pH-dependent release profile. Slower release rates were observed under physiological pH (7.4) conditions, and accelerated release in acidic environments (pH 4.8). The study demonstrated the efficacy of the nanocrystal carrier system in delivering DOX to target cells, as evidenced by the significant inhibition of MG-63 cells. These findings highlight the potential of calcium carbonate nanocrystals as biocompatible carriers for controlled drug delivery, offering promising implications for the clinical treatment of OS and other malignancies^^80^^.

Calcium-Carbonate Nanocrystals - In this study, two types of nanoparticles: pure calcium HA and cerium-substituted hydroxyapatite (Ce-HA) with different cerium concentrations were synthesized. Various analytical techniques were employed to characterize the nanoparticles' structure and properties. Cerium-doped nanoparticles exhibited potent antibacterial and antifungal effects and bioactivity in simulated body fluid. They demonstrated sustained release of the anticancer drug DOX over 28 days, with statistical significance observed in release kinetics between Ce-HA and HA nanoparticles. Additionally, cytotoxicity assays on bone cancer cells indicated promising anticancer activity of DOX-loaded Ce-HA nanoparticles, especially at higher cerium levels. These findings suggest the potential of Ce-HA nanoparticles for targeted drug delivery in bone cancer therapy, warranting further investigation^^81^^.

Hybrid Nanogels for Co-Delivery of CDDP and DOX - This study used a preparation of cisplatin (CDDP)-crosslinked hyaluronic acid (HA) nanogel (^^CDDP^^HANG) for effective drug delivery. Here, CDDP acts as a crosslinker and an ancillary anticarcinogen to prevent premature release, enhance circulating periods, and reduce the adverse effects of DOX. Cationic DOX is incorporated into the nanoparticle through the electronic interaction with anionic HA, obtaining HA/DOX. Furthermore, Confocal laser scanning microscopy and flow cytometry are used to visualize intracellular DOX activity in mouse osteosarcoma K7 cells. It concluded that small-molecule DOX could pass through the cell membrane by fast diffusion and show a robust fluorescent intensity than that of the ^^CDDP3.3^^HANG/DOX__5.4__ group. However, the diffusion pathway fails to thrive for a longer duration, marking the nano gel encapsulated DOX’s endocytosis pathway as favorable for long-term therapy. H&E staining of multiple organs was performed to evaluate the long-term toxicity of ^^CDDP3.3^^HANG/DOX__5.4__. This revealed no apparent morphological changes in the spleen and lung, reduced pathological changes, and necrosis of the heart. In contrast, mice treated with free DOX plus CDDP showed critical texture deranging and fracture of the muscle fibers and moderate hepatocyte and hepatic lobule damage. Therefore, negligible organic injury of ^^CDDP3.3^^HANG/DOX__5.4__ potentially proved its reduced long-term toxicity. Overall, CDDP crosslinked DOX-loaded nano gel exhibited synergistic antitumor effects, optimized biodistribution, and reduced side toxicity^^82^^.

Despite the promising preclinical data on novel drug delivery systems, a significant translational gap exists between these findings and their widespread clinical application (Table 2). While a wide array of novel delivery systems, including polymer-based nanoparticles, light-responsive nano-micelles, and hydrogels, have shown significant preclinical promise in enhancing drug delivery and anti-tumor efficacy, their progression to human trials remains limited. Among the discussed delivery systems, PEG-LD stands out as the most promising candidate. Unlike the other platforms, PEG-LD has successfully advanced to a Phase I clinical trial. This demonstrates an acceptable safety profile and a maximum tolerated dose when combined with cisplatin in patients with metastatic and recurrent OS. These results highlight PEG-LD’s potential for real-world application and validate the principle of using targeted delivery to enhance therapeutic efficacy while minimizing systemic toxicities. Polymer-based hydrogels offer localized, sustained release but remain in preclinical development, possibly due to challenges related to limited mechanical strength and difficulties in controlling degradation rates^^83^^. Nanoparticle-based systems, including light-responsive micelles and cerium-substituted hydroxyapatite, provide precise targeting and tunable release profiles but face significant manufacturing, regulatory, and cost-related hurdles^^84,85^^. Collectively, PEG-LD and hybrid nanogels appear the most promising for near-term clinical translation, whereas advanced nanoparticles may shape next-generation strategies once scale-up and safety concerns are resolved.

**Table 2:** Benefits and Limitations of DOX Delivery Systems in Osteosarcoma Treatment

Delivery System	Benefits	Limitations
Polymer-Based Systems (e.g., hydrogels, PLGA-PEG-PLGA)	- Sustained and controlled DOX release.- Minimally invasive (injectable, thermosensitive hydrogels).- Possibility of co-delivery with other drugs or genes for synergistic effects.	- Mostly limited to the preclinical stage.- Potential variability in degradation and release kinetics in vivo.- Long-term safety and biocompatibility are still uncertain.
Hydroxyapatite (HA)-Based Systems	- Strong DOX binding (up to 91.8%).- pH-dependent release enables targeted tumor delivery.- Promotes bone regeneration and osteogenesis.	- Evidence primarily from animal models.- Limited data on systemic toxicity in humans.- Translational gap from preclinical to clinical use.
Pegylated Liposomal DOX (PEG-LD)	- Reduced cardiotoxicity compared to free DOX.- Established maximum tolerated dose in Phase I trial.- Promising safety profile and some clinical activity in OS patients.	- Response rate still modest (13.3%).- Myelosuppression and stomatitis remain common adverse effects.- Requires further validation in larger Phase II/III trials.
Platinum Nanoparticles (PtNPs) + DOX	- Synergistic anticancer activity via oxidative stress and apoptosis.- Lower DOX doses needed when combined with PtNPs.	- Mechanistic understanding is still incomplete.- Potential nanoparticle-related systemic toxicity has not been fully studied.- Preclinical data only.
Calcium Phosphate–Phosphorylated Adenosine (CPPA) Microspheres	- High DOX encapsulation efficiency (~42.3%).- pH-responsive release ensures tumor-specific delivery.- Enhances osteogenesis along with anticancer effects.	- Complex preparation methods may limit scalability.- Limited in vivo safety/efficacy data.- No clinical validation yet.
Nanoparticle-Based Systems (Ag2O-MBG, CaCO3, Ce-HA, light-responsive micelles, hybrid nanogels)	- High encapsulation efficiency and controlled, pH-dependent release.- Potential for tumor-specific targeting (EPR effect, light-triggered release).- Some systems show synergistic effects with CDDP.- Reduced long-term toxicity in nanogel-based systems.	- All still preclinical; lack of human trials.- Manufacturing and reproducibility challenges.- Risk of unforeseen immunological or systemic toxicities.

## 6. Challenges and Future Directions

The development of novel DOX delivery systems represents a significant progress in cancer therapy. However, their clinical translation faces several limitations^^86,87^^. Patients often require administration of large amounts of “empty” carrier material, with DOX comprising only 5-10% of the total weight. This not only raises toxicity concerns but also increases costs and reduces efficiency^^87^^. Additionally, premature leakage of DOX from carriers while circulating in the bloodstream can result in systemic toxicity, undermining the very purpose of nanocarrier-based delivery^^86,87^^.

Inorganic nanoparticles pose further safety challenges due to their long half-lives and lack of biodegradability, while the synthesis of complex carriers, such as dendrimers, is expensive and difficult to scale^^86,87^^. The size and surface characteristics of carriers can also hinder deep tumor penetration, leaving hypoxic tumor cores untreated and contributing to relapse^^83^^. Drug resistance mechanisms, such as the overexpression of efflux pumps like P-glycoprotein, can actively expel DOX from cancer cells, reducing its efficacy^^88^^.

From a manufacturing perspective, producing nanocarriers with consistent size, drug loading, and purity on a commercial scale is highly challenging and costly^^87^^. Repeated administration can trigger immune recognition, leading to the Accelerated Blood Clearance phenomenon and reducing therapeutic efficacy over time^^87^^. The complexity of these novel formulations further drives up the cost of treatment, raising concerns about accessibility^^86,87^^.

Future research should focus on developing smart, stimuli-responsive carriers that release DOX only in response to tumor-specific triggers, such as low pH, high enzyme levels, or elevated ROS, minimizing off-target effects and systemic toxicity^^87^^. Designing biodegradable polymers and lipids with higher drug loading capacities can address low drug-to-carrier ratios and improve formulation stability^^87,89^^. Additionally, scalability and clinical relevance should be prioritized through human-relevant disease models and reproducible synthesis methods to facilitate large-scale production^^87,90^^. Achieving the transformation of DOX from a broadly cytotoxic agent into a precision therapeutic will require simultaneous advances in biological efficacy, manufacturing scalability, and patient safety.

## 7. Conclusion

DOX serves as the foundational therapy in osteosarcoma management. However, its therapeutic potential to reach tumor-specific concentrations following post-systemic administration is limited, owing to its life-threatening cardiotoxicity. Advances in targeted delivery platforms, particularly polymer- and nanoparticle-based systems, demonstrate promising potential to enhance drug selectivity, reduce systemic toxicity, and improve therapeutic outcomes. Future progress depends on integrating these novel delivery strategies with deeper insights into resistance mechanisms and molecular pathways. This not only optimizes treatment efficacy but also reduces the need for high-dose administration. Ultimately, such innovations hold the potential to improve both survival rates and quality of life for patients with osteosarcoma.
